# Tropism and Replication Competence of Cattle Influenza A(H5N1) Genotype B3.13 Virus in Human Bronchus and Lung Tissue

**DOI:** 10.3201/eid3205.251926

**Published:** 2026-05

**Authors:** Kenrie P.Y. Hui, John C.W. Ho, Ka-Chun Ng, Richard J. Webby, Malik Peiris, John M. Nicholls, Michael C.W. Chan

**Affiliations:** Centre for Immunology & Infection, Hong Kong, China (K.P.Y. Hui, J.C.W. Ho, M. Peiris, M.C.W. Chan); School of Public Health, Li Ka Shing Faculty of Medicine, University of Hong Kong, Hong Kong (K.P.Y. Hui, K.-C. Ng, M. Peiris, M.C.W. Chan); St. Jude Children’s Research Hospital, Memphis, Tennessee, USA (R.J. Webby); School of Clinical Medicine, Li Ka Shing Faculty of Medicine, University of Hong Kong, Hong Kong (J.M. Nicholls)

**Keywords:** Influenza, respiratory infections, zoonoses, viruses, cattle influenza, H5N1, ex vivo, cytokine, inflammatory, human respiratory tract, Hong Kong

## Abstract

In 2024, influenza A(H5N1) genotype B3.13 viruses emerged from cattle and caused mild spillover infections in humans. Using human bronchus and lung tissue, we evaluated tropism, replication, and pathogenesis of 2 cattle influenza isolates. Those viruses showed moderate replication competence and induced robust proinflammatory responses, suggesting potential risk for human health.

Highly pathogenic avian influenza (HPAI) H5N1 viruses remain a major global health concern, particularly because of sporadic spillover into mammals (*1*). HPAI A(H5N1) clade 2.3.4.4b viruses entered the United States through a trans-Atlantic introduction in late 2021, after which extensive reassortment among migratory birds produced the B3.13 and D1.1 genotypes. Those variants have spread widely, driving outbreaks in livestock and causing occasional human infections (*2*,*3*). Beginning in 2024, H5N1 clade 2.3.4.4b viruses were detected in dairy cattle across multiple US states, and those infections were linked to mild zoonotic cases in humans (*4*,*5*). To assess the health risks of emerging cattle-origin influenza viruses, we examined tropism, replication, receptor use, and innate immune responses of cattle H5N1 viruses in human respiratory tract explants.

## The Study

We investigated newly emerged cattle influenza A(H5N1) genotype B3.13 virus strains A/dairy_cow/Ohio/B24OSU-439/2024 (H5N1/439) and A/dairy_cow/Texas/98638/2024 (H5N1/98638) in human bronchial and lung tissue cultures (Appendix). In brief, we obtained nonmalignant tissue cultures from patients who underwent elective surgery and consented to tissue use (Appendix). We used the RNeasy Micro Kit (QIAGEN, https://www.qiagen.com) to extract total RNA, according to manufacturer instructions, then reverse transcribed extracted RNA by using the PrimeScript RT Reagent Kit (TaKaRa Bio, Inc., http://www.takara-bio.com). We fixed the explant tissues for immunohistochemical staining of influenza viral proteins.

**Figure 1:**
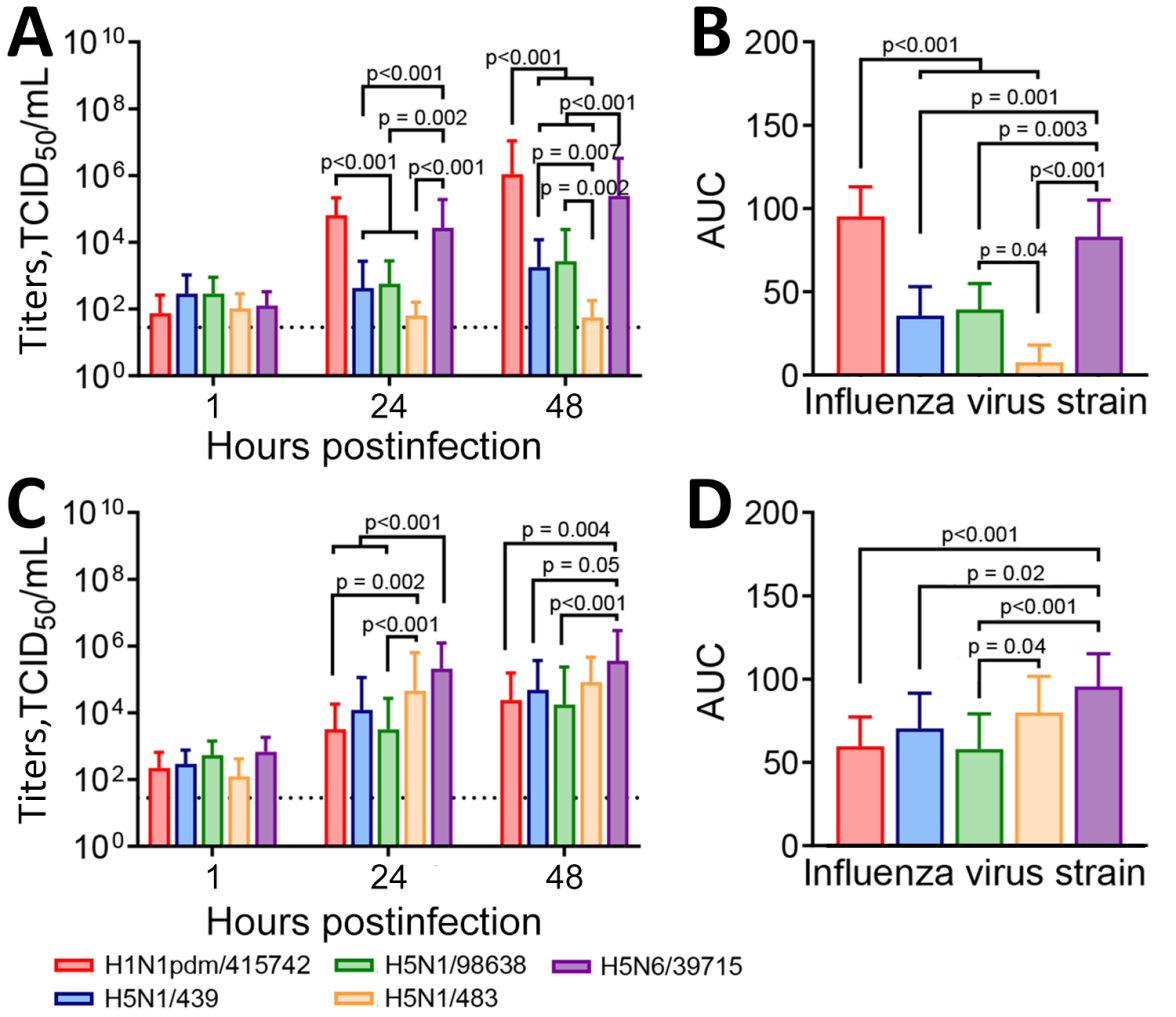
Viral replication kinetics in a study of ex vivo tropism and replication competence of cattle influenza A(H5N1) genotype B3.13 virus in human bronchus and lung tissue. A, B) Mean virus titers for bronchus tissues; C, D) mean virus titers for lung tissues. A, C) Culture supernatants of the infected human respiratory tissue explants were harvested at 1, 24, and 48 hours postinfection and viral titers determined by TCID_50_ assays; dashed line indicates assay detection limit. Statistical significance was calculated by using 2-way analysis of variance with Tukey post-hoc test. Error bars indicate SD. B, D) AUC values calculated from influenza virus titers 24–48 hours postinfection are shown. Statistical significance was calculated by using 1-way analysis of variance with Tukey post-hoc test. Error bars indicate SD. AUC, area under the curve; TCID_50_, 50% tissue culture infectious dose.

We compared explant cultures with 3 historical human isolates: H5N1/483, H5N6/39715, and H1N1pdm/415742 (Appendix Tables 1, 2). The historical virus strains showed different tropisms and area under the curve (AUC) levels to the explant tissue cultures at 24–48 hours postinfection (hpi). In bronchus tissues, H1N1pdm/415742 replication was highest, followed by H5N6/39715 (Figure 1, panels A, B). The H5N1/439 and H5N1/98638 cattle isolates showed substantially lower replication competence at 48 hpi than H1N1pdm/415742 and H5N6/39715 but much higher competence than the H5N1/483 avian isolate, which replicated poorly in the human bronchial tissues. In human lung tissues, H5N6/39715 replicated to the highest viral titers and AUC values at 24 hpi, followed by H5N1/483 (Figure 1, panels C, D). Both cattle isolates replicated to similar levels as H1N1pdm/415742, but isolate H5N1/439 had a slightly higher replication trend than H5N1/98638. The cattle-origin viruses replicated to lower titers than HPAI H5N1 but similar to H1N1pdm/415742 in human lung tissues, which aligns with other reports in human lung organoids (*6*). However, cattle-origin H5N1 genotype B3.13 virus has lower replication than genotype D1.1 in human nasal and airway organoids (*7*). Replication competence in human respiratory tissues might be contributed by the 631L of polymerase basic protein 2 (Appendix Table 2), which promotes polymerase activity in human cells (*8*). In addition, the cattle-origin H5N1 viruses replicated to higher titers than avian H5N1 viruses in bronchial tissues, implying that the cattle-origin H5N1 viruses might be more transmissible than HPAI H5N1 viruses.

**Figure 2:**
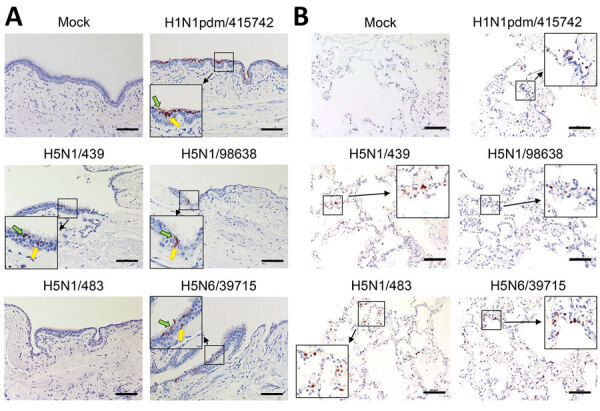
Immunohistochemical stain of nucleoprotein from samples in a study of tropism and replication competence of cattle influenza A(H5N1) genotype B3.13 virus in human bronchus and lung tissue. Formalin-fixed, paraffin-embedded sections of human bronchus (A) and lung (B) tissue explants at 48 hours postinfection are shown. Cells positive for influenza A nucleoprotein are indicated by red-brown color. Green arrows indicate ciliated cells; yellow arrows indicate nonciliated cells. Images are representatives of 3 separate donors. Cattle influenza A(H5N1) genotype B3.13 virus strains A/dairy_cow/Ohio/B24OSU-439/2024 (H5N1/439) and A/dairy_cow/Texas/98638/2024 (H5N1/98638) are compared with historical human isolates of highly pathogenic avian influenza strains H5N1/483, H5N6/39715, and H1N1pdm/415742 (Appendix Tables 1, 2). Scale bars indicate 100 μm.

Immunohistochemical staining revealed the tissue and cellular tropism of cattle-origin H5N1 viruses. We found nucleoprotein (NP)–positive cells from H1N1pdm/415742 virus and to a lesser extent H5N6/39715 in the bronchial epithelium, infecting ciliated and nonciliated epithelial cells (Figure 2, panel A). In comparison, we noted moderate levels of NP-positive cells from cattle H5N1/439 and H5N1/98638 viruses in ciliated and nonciliated epithelial cells but identified no NP-positive cells from avian-origin H5N1/483 in the bronchial sections. In the lung sections, H5N1/483 and H5N6/39715 demonstrated the most extensive infections, followed by H5N1/439, H5N1/98638, and H1N1pdm/415742 (Figure 2, panel B). Together with the viral replication data, those results imply that cattle-origin H5N1/439 and H5N1/98638 viruses possess moderate replication capacity in upper and lower airways and are better adapted to human hosts than avian H5N1/483.

To test virus agglutination, we conducted selective desialylation on turkey red blood cells (TRBCs). For controls, we used H1N1pdm/415742, known for binding to α(2–6)-linked sialic acid (SA), and H5N1/483, known for binding to α(2,3)-linked SA. Treating TRBCs with Sialidase S (Agilent, https://www.agilent.com), which preferentially cleaves the α(2,3)-linked SA, prevented hemagglutination of avian H5N1/483 but did not affect H1N1pdm/415742, H5N1/439, or H5N1/98638 isolates (Table). Conversely, treating with Sialidase C (Agilent), which cleaves α(2,3) and α(2–6) linkages, and Sialidase A (Agilent), which cleaves α(2,3), α(2–6), α(2–8), and α(2–9) linkages, prevented hemagglutination of all the influenza strains. Those results suggest that the cattle-origin H5N1/439 and H5N1/98638 isolates resemble H1N1pdm/415742 and acquired at least partial affinity to α(2–6)-linked SA, which differs from the avian H5N1/483 virus, indicating that the 2 emergent cattle viruses could have a higher host adaption to humans. 

**Table:** Effects of desialylation on virus hemagglutination in a study of ex vivo tropism and replication competence of cattle influenza A(H5N1) genotype B3.13 virus in human bronchus and lung tissue*

Influenza virus strain	Desialylation treatments			
	Untreated	Sialidase S	Sialidase C	Sialidase A
A(H1N1)pdm09				
A/Hong Kong/415742/2009	16	16	0	0
Human A(H5N1)				
A/Hong Kong/483/1997	8	0	0	0
Cattle A(H5N1)				
A/dairy_cow/Ohio/B24OSU-439/2024	8	8	0	0
A/dairy_cow/Texas/98638/2024	8	8	0	0

*Studies performed in 0.5% turkey red blood cells. The reciprocal of hemagglutination titers is noted. Experiments were performed in triplicate and led to identical results. Substrate specificity of sialidases (all Agilent, https://agilent.com) are as follows: α(2,3)-linked sialic acid for sialidase S; α(2,3)- and α(2–6)-linked sialic acids for sialidase C; and α(2,3)-linked, α(2–6)-linked, α(2–8)-linked, and α(2–9)-linked sialic acids for sialidase A.

A/Texas/37/2024 and other bovine-origin H5N1 isolates have shown dual binding affinity to α(2,3)- and α(2–6)-linked SA (*7**–**10*). However, multiple studies reported contradictory findings on the α(2–6)-linked receptor-binding specificity for different bovine-origin H5N1 isolates (*11*–*13*). One study (*13*) reported that A/bovine/Ohio/B24OSU-432/2024, which has a hemagglutinin amino acid sequence identical to that of virus examined elsewhere (*10*), preferentially binds to avian-type α(2,3) sialoside receptors. Genotypic differences cannot explain the binding affinity discrepancies (Appendix Tables 3, 4), but they might be explained by technical differences in the assays and origins of virus propagation in mammalian cells or embryonated eggs, which affect glycosylation of progeny viruses and hence receptor-binding specificity. Moreover, the binding affinity of H5N1/439 and H5N1/98638 aligned with their higher replication competences in bronchial tissues than the avian-origin H5N1/483 virus, which predominantly expresses an α(2–6)-linked SA. Those findings suggest that the 2 cattle-origin H5N1 viruses have a higher potential for human-to-human transmission than avian-origin H5N1.

**Figure 3:**
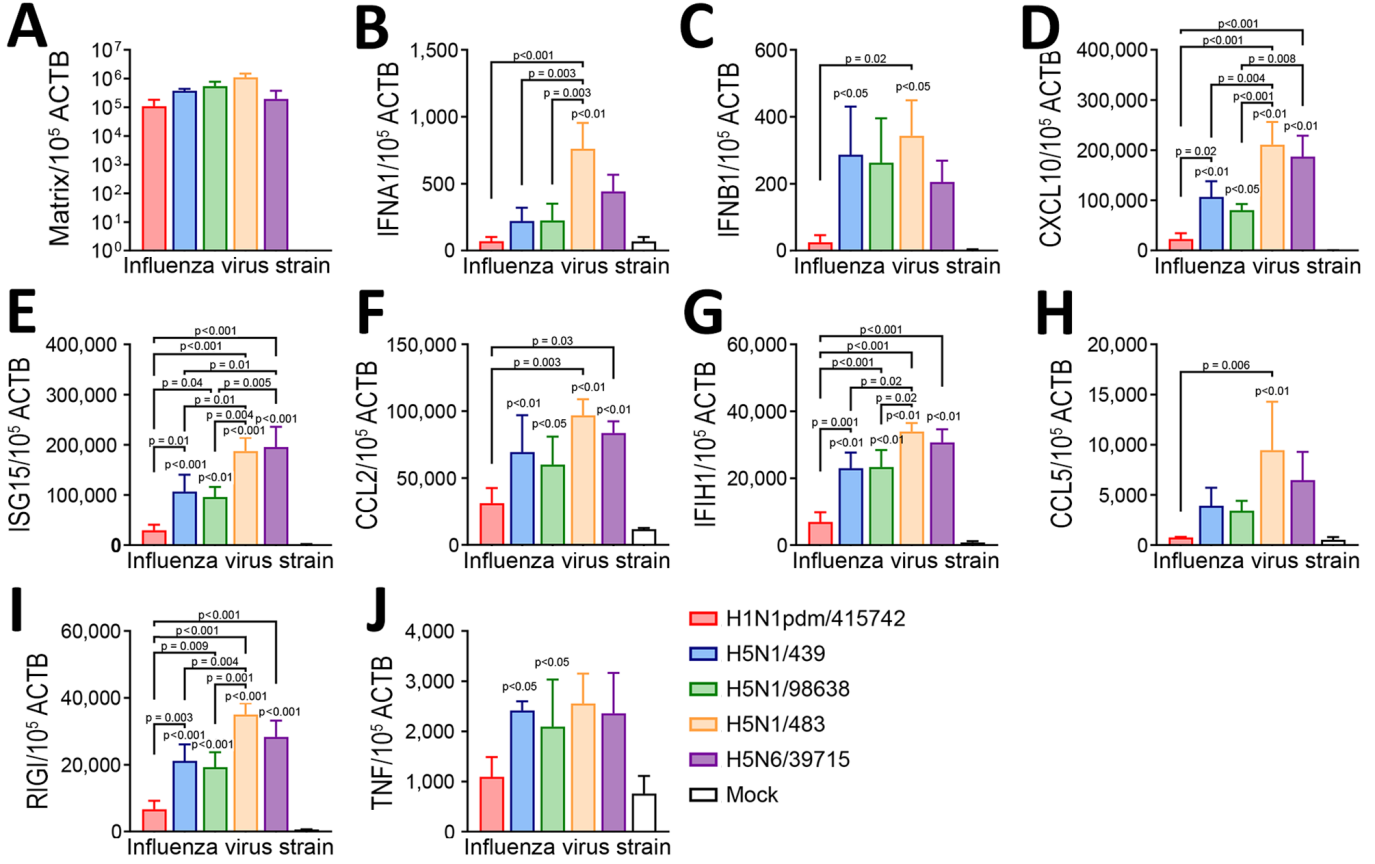
Cytokine and chemokine mRNA expressions in a study of tropism and replication competence of cattle influenza A(H5N1) genotype B3.13 virus in human lung tissue. A) Matrix gene; B) IFNA1; C) IFNB1; D) CXCL10; E) ISG15; F) CCL2; G) IFIH1; H) CCL5; I) RIGI; J) TNF. Expressions measured at 48 hours postinfection. Data were pooled from >3 independent experiments. Bars indicate mean, whiskers indicate SD. Statistical significance was calculated by using 1-way analysis of variance with Tukey posthoc test. Statistical significance is in comparison with mock. ACTB, beta-actin; CCL, C-C motif chemokine ligand; CXCL, C-X-C motif chemokine ligand; IFIH, interferon induced with helicase C domain; IFNA, interferon alpha; IFNB, interferon beta; ISG, interferon-stimulated gene; RIGI, retinoic acid–inducible gene; TNF, tumor necrosis factor.

Because illness severity of H5N1 infection is associated with induction of proinflammatory cytokines (*14*), we compared innate immune responses among the 5 viruses in human lung tissue. HPAI H5N1/483 infection induced substantially higher mRNA expressions of interferon alpha 1 (IFNA1), C-X-C motif chemokine ligand 10 (CXCL10), interferon-stimulated gene 15 (ISG15), interferon induced with helicase C domain 1 (IFIH1), and retinoic acid–inducible gene I (RIGI) than cattle-origin H5N1/439 and H5N1/98638. However, we observed a trend of elevated C-C motif chemokine ligand 2 (CCL2) and ligand 5 (CCL5) expression (Figure 3). H5N6/39715 infection induced substantially higher levels of ISG15, CCL2, CXCL10, IFIH1, and RIGI than H1N1pdm/415742, but H5N6/39715 had higher CXCL10 induction than H5N1/98638 and elevated ISG15 compared with H5N1/98638 and H5N1/439. 

The 2 cattle-origin H5N1 isolates induced similar immune responses and substantially higher levels of IFNB1, ISG15, tumor necrosis factor, CCL2, CXCL10, IFIH1, and RIGI over the mock-infected tissues (Figure 3). Proinflammatory gene induction by H5N1/439 and H5N1/98638 viruses had higher trends than proinflammatory induction by H1N1pdm/415742, and substantial differences in ISG15, CXCL10, IFIH1, and RIGI. Prior animal studies demonstrated that H5N1/439 and H5N1/98638 and a human isolate were lethal to mice and ferrets (*8*,*11*). However, we detected lower immune response induction and fewer proinflammatory cytokines in human lung tissues, consistent with other studies (*6*), suggesting that those factors might contribute to reduced pathogenicity of cattle-origin H5N1 compared with avian H5N1.

## Conclusions

Viral titers and influenza NP-positive cells demonstrated that cattle-origin H5N1/439 and H5N1/98638 strains are better adapted to human upper airway tissues than avian H5N1/483 and have similar replication abilities as H1N1pdm/415742 in human lung explants. The ability to bind α(2–6)-linked SA further indicates a shift of receptor affinities that are more compatible with upper respiratory tissues. Innate immune responses of H5N1/439 and H5N1/98638 viruses in human lung tissue fell between those triggered by H1N1pdm/415742 and H5N1/483 viruses, indicating that cattle H5N1 viruses could pose a human health risk. Defining how these strains infect human tissues and shape immune responses is critical for anticipating outbreaks and reducing zoonotic transmission risks. Because influenza viruses continually evolve across diverse avian and mammalian hosts, sustained research and surveillance remain essential to prevent human infections. 

Ethics approval of the use of human tissues was granted by the institutional review board of University of Hong Kong (approval no. UW 20–862).

Appendix Additional information on tropism and replication competence of cattle influenza A(H5N1) genotype B3.13 virus in human bronchus and lung tissue.
