# Supplementary material for: Tropism and Replication Competence of Cattle Influenza A(H5N1) Genotype B3.13 Virus in Human Bronchus and Lung Tissue
Source: Emerg Infect Dis. 2026 May;32(5):800–4. doi: 10.3201/eid3205.251926 (PMC13175097; doi:10.3201/eid3205.251926)
Supplement: Appendix — Additional information on tropism and replication competence of cattle influenza A(H5N1) genotype B3.13 virus in human bronchus and lung tissue. [file 25-1926-Techapp-s1.pdf]

Article DOI: https://doi.org/10.3201/eid3205.251926

EID cannot ensure accessibility for supplementary materials supplied by authors. Readers who have difficulty accessing supplementary content should contact the authors for assistance.

Tropism and Replication Competence of Cattle Influenza A(H5N1) Genotype B3.13 Virus in Human Bronchus and Lung Tissue

Appendix

Materials and Methods

Viruses

Two newly emerged H5N1 influenza A virus strains in cattle including A/dairy_cow/Ohio/B24OSU-439/2024 (H5N1/439) and A/dairy_cow/Texas/98638/2024 (H5N1/98638) were studied, along with three historical human isolates including the highly pathogenic avian influenza H5N1 A/Hong Kong/483/1997 (H5N1/483), H5N6 A/Guangzhou/39715/2014 (H5N6/39715) and the pandemic H1N1 A/Hong Kong/415742/2009 (H1N1pdm/415742). The GISAID accession numbers of these viruses are listed in Appendix Table 1. The viruses were propagated and titrated with Madin-Darby canine kidney (MDCK) cells. All experiments involving live viruses were carried out in the biosafety level 3 facility at The University of Hong Kong Special Administrative Region, People’s Republic of China.

Ex vivo human bronchus and lung tissue infection

Human respiratory tract explants were derived and infected as previously described (*1*,*2*). Briefly, non-malignant bronchus and lung tissues were obtained from consented patients who underwent elective surgery at the Department of Surgery, Queen Mary Hospital. Tissues were removed for clinical care and surplus for diagnostic requirements. On the same day of tissue removal, the tissues were fragmented into similarly sized pieces with scalpels and infected by immersing in virus inoculum at 1×10^6 pfu/mL of influenza viruses at 37°C for 1 h. The infected tissues were washed with phosphate buffered saline, placed in fresh F-12K nutrient mixture with L-glutamine (GIBCO) and 1% antibiotics in 24-well tissue culture plates and incubated at 37°C with 5% CO_2. Mock-infected tissues were used as controls. Culture supernatants were collected at 1–48 hpi to assess the viral titers. Tissues were harvested at endpoints for gene expression quantification or fixed in 10% formalin for immunohistochemistry staining. Ethics approval of the use of human tissues was granted by the institutional review board of The University of Hong Kong and the Hospital Authority (IRB approval no: UW 20–862).

Viral titration

Virus titers in culture supernatants were determined by 50% tissue culture infectious dose ($TCID_{50}$) assay. Briefly, culture supernatants were serially diluted from 0.5- to 7-log and added to MDCK cells on 96-well culture plates in quadruplicate for 72 h incubation. The endpoints of dilution with cytopathic effects to MDCK cells in 50% of the inoculated wells were used to calculate virus titers using the Spearman-Kärber method. Area-under-the-curve (AUC) values were calculated from the viral titers from different time points indicated in the y-axis.

RNA extraction and quantitative PCR

Total RNA was extracted using RNeasy Micro Kit (Qiagen) according to the manufacturer's instructions. Extracted RNA was reverse transcribed using PrimeScript RT Reagent Kit (TaKaRa). Viral and cell gene expressions were quantified by qPCR with the corresponding gene primers (Appendix Table 5) and SYBR Premix Ex *Taq*II (TaKaRa). Gene copies were evaluated with the respective standards and normalized with β-actin (ACTB) gene levels.

Desialylation-hamagglutination assay

The receptor specificity of influenza virus haemagglutinin protein was tested with the desialylation of Turkey red blood cells (TRBCs, Lampire). Desialylation was achieved by treating 0.5% TRBCs with either Sialidase S (Agilent) that preferentially cleaves α(2,3)-linked sialic acid, Sialidase C (Agilent) that cleaves both α (2,3)- and α (2–6)-linked sialic acids, or Sialidase A (Agilent), which cleaves α(2,3,6,8,9)-linked sialic acids. Influenza viruses were 2-fold serially diluted and were mixed with equal volumes of the treated or untreated TRBCs in 96-well plates. The plates were incubated at room temperature for 2 h and the highest dilutions of influenza viruses that gave haemagglutination were compared among desialylation treatments.

Immunohistochemistry staining

The ex vivo human tissues fixed with 10% formalin were stained for influenza viral protein. Briefly, the tissues were embedded in paraffin blocks, sectioned onto glass slides, deparaffinized for antigen retrieval, blocked for endogenous peroxidase activity, and then incubated with primary antibody against influenza A virus nucleoprotein (mouse monoclonal HB65) followed by horseradish peroxidase-conjugated secondary antibody (Vector Laboratory). The staining was developed using NovaRED Substrate Kit (Vector Laboratory) and

counterstained for cell nuclei with Mayer's Hematoxylin. The stained tissue sections were imaged using the ECLIPSE Ti-S microscope (Nikon Instruments).

Statistical analysis

Experiments were performed using five different donors of bronchus and lung tissues, respectively, each in duplicates or triplicates. Results were presented as the mean values±standard deviation, with the differences considered to be significant at $p < 0.05$. Statistical analyses were performed with one-way or two-way ANOVA with Tukey's multiple comparison test using GraphPad Prism version 10 (GraphPad Software).

References

1. Nicholls JM, Chan MC, Chan WY, Wong HK, Cheung CY, Kwong DL, et al. Tropism of avian influenza A (H5N1) in the upper and lower respiratory tract. Nat Med. 2007;13:147–9. PubMed https://doi.org/10.1038/nm1529

2. Chan MC, Chan RW, Yu WC, Ho CC, Yuen KM, Fong JH, et al. Tropism and innate host responses of the 2009 pandemic H1N1 influenza virus in ex vivo and in vitro cultures of human conjunctiva and respiratory tract. Am J Pathol. 2010;176:1828–40. PubMed https://doi.org/10.2353/ajpath.2010.091087

Appendix Table 1. Molecular features associated with viral pathogenicity, transmissibility and antiviral resistance

					HA									
					Glycosylation motif	Receptor binding site								
Virus	Accession no.	Subtype	Host	Clade	174–176	150–153 (del 152)	203	206	209	212	240	242	240–244	Cleavage site
A/Hong_Kong/483/1997	EPI_ISL_964	H5N1	Human	0	NST	SGVSS	D	E	K	Q	N	Q	NGQSG	RERRRKKRG
A/Guangzhou/39715/2014	EPI_ISL_175335	H5N6	Human	2.3.4.4	NDT	LGVSA	N	E	N	K	N	Q	NGQRG	RERRR-KRG
A/dairy_cow/Ohio/B24OSU-439/2024	EPI_ISL_19178083	H5N1	Cattle	2.3.4.4b	NDA	LGVSA	N	E	N	K	N	Q	NGQRG	REKRR-KRG
A/dairy_cow/Texas/98638/2024	EPI_ISL_20053953	H5N1	Cattle	2.3.4.4b	NDA	LGVSA	N	E	N	K	N	Q	NGQRG	REKRR-KRG
A/Hong_Kong/415742Md/2009	EPI_ISL_87431	H1N1	Human	6B.1	ENS	KGVTA	T	D	K	Q	R	Q	RGQEG	IQ----SRG

*H3 numbering (A/Achi/2/1968).

Appendix Table 2. Molecular features associated with viral pathogenicity, transmissibility and antiviral resistance

	NA†						PA				PB1	PB2											NS1				M2
Virus	60–71 delete	119	222	246	274	292	100	336	356	409	368	147	158	271	339	340	588	591	627	631	701	702	42	92	205	210	31
A/Hong_Kong/483/1997	Yes	E	I	S	H	R	V	M	K	S	I	M	E	T	K	R	A	Q	K	M	D	K	S	E	S	G	S
A/Guangzhou/39715/2014	No	E	I	A	H	R	V	L	K	S	I	T	E	T	T	K	T	Q	K	M	D	K	S	E	S	G	S
A/dairy_cow/Ohio/B24OSU-439/2024	No	E	I	S	H	R	V	L	K	S	I	I	E	T	K	R	A	Q	E	L	D	K	S	D	S	G	S
A/dairy_cow/Texas/98638/2024	No	E	I	S	H	R	V	L	K	S	I	I	E	T	K	R	A	Q	E	L	D	K	S	D	S	G	S
A/Hong_Kong/415742Md/2009	No	E	I	S	H	R	V	M	R	N	I	T	E	A	K	K	T	R	E	M	D	K	S	D	S	G	N

*Yellow indicates mammalian adaptation.

†N2 numbering (A/Tokyo/3/67).

Appendix Table 3. Identity and amino acid comparison of cattle-H5N1/439 and cattle-H5N1/98638

Gene	Virus	Subtype	Identity	Difference in amino acid	
HA*	439	H5N1	100.0%		
	98638	H5N1			
				34	71
NA†	439	H5N1	92.3%	V	N
	98638	H5N1		I	S
MP1	439	H5N1	100.0%		
	98638	H5N1			
MP2	439	H5N1	100.0%		
	98638	H5N1			
				85	
NP	439	H5N1	99.8%	A	
	98638	H5N1		T	
				76	
NS1	439	H5N1	99.6%	A	
	98638	H5N1		T	
				86	
NEP	439	H5N1	99.2%	T	
	98638	H5N1		I	
				598	
PA	439	H5N1	99.9%	A	
	98638	H5N1		T	
PB1	439	H5N1	100.0%		
	98638	H5N1			
				400	
PB2	439	H5N1	99.9%	T	
	98638	H5N1		A	

*H5 numbering (A/Hong_Kong/483/1997).

†N1 numbering (A/Hong_Kong/483/1997).

Appendix Table 4. Identity and amino acid comparison of the HA of 4 H5N1 viruses

Virus	Subtype	Identity	Difference in amino acid
HA gene			339
439	H5N1	99.8%	S
98638	H5N1		S
A/dairy_cow/New_Mexico/A240920343–93/2024*	H5N1		N
A/Texas/37/2024#	H5N1		S

*H3 numbering (A/Aichi/2/1968).

Appendix Table 5. The nucleotide sequences of primers used for quantitative PCR.

Gene	Primer sequence (5′-3′)
ACTB	F: TGGATCAGCAAGCAGGAGTATG
	R: GCATTTGCGGTGGACGAT
Influenza M-gene	F: CTTCTAACCGAGGTCGAAACG
	R: GGCATTTTGGACAAAKCGTCTA
IFNA1	F: AGAAGGCTCCAGCCATCTCTGT
	R: TGCTGGTAGAGTTCGGTGCAGA
IFNB1	F: CAACTTGCTTGGATTCCTACAAAG
	R: TGCCACAGGAGCTTCTGACA
CXCL10	F: ATTATTCCTGCAAGCCAATTTTG
	R: TCACCCTTCTTTTTCATTGTAGCA
ISG15	F: CAAATGCGACGAACCTCTGA
	R: CCGCTCACTTGCTGCTTCA
CCL2	F: CAAGCAGAAGTGGGTTCAGGAT
	R: TCTTCGGAGTTTGGGTTTGC
IFIH1	F: TCACAAGTTGATGGTCCTCAAGT
	R: CCTTCTCCAGATTTGGCTGAAC
CCL5	F: CTTTGCCAGGGCTCTGTGA
	R: GCAGTGTTCCTCCCCTCCTT
RIGI	F: GCGTCAGTGATAGCAACAGTCAA
	R: TCGCTAATCCGTGATTCCACTT
TNF	F: GCAGGTCTACTTTGGGATCATTG
	R: GCGTTTGGGAAGGTTGGA
